# Abdominal pregnancy as a cause of hemoperitoneum

**DOI:** 10.4103/0974-2700.55342

**Published:** 2009

**Authors:** Sheikh Muzamil Shafi, Misbha Afsheen Malla, Parvaiz Ahmed Salaam, Omer Shareef Kirmani

**Affiliations:** Postgraduate Department of Surgery, SMHS Hospital, Srinagar, India

**Keywords:** Abdominal pregnancy, ectopic pregnancy, heterotopic pregnancy

## Abstract

The coexistence of intrauterine and extrauterine pregnancy, the heterotopic pregnancy, is a rare obstetric phenomenon. The preoperative diagnosis of this condition is very difficult; leading to a higher maternal morbidity and fetal loss. We experienced a case of intrauterine pregnancy and ruptured abdominal pregnancy implanted on the illeocaecal region in a 26-year-old primiparous woman. She was clinically misdiagnosed as a case of ruptured ectopic pregnancy, but ultrasonography showed it to be a case of heterotopic pregnancy. Subsequently, the patient was subjected to laparotomy and the ruptured abdominal pregnancy was evacuated. She continued with the intrauterine pregnancy till term and delivered a healthy female baby. Although this condition is unusual, any general surgeon in the emergency department must be aware of this complication and its management, which is often initially misdiagnosed.

## INTRODUCTION

Abdominal pregnancy is a rare event but is associated with significant morbidity and mortality. The incidence varies widely with geographical location, degree of antenatal attendance, level of medical care and socioeconomic status.[1] It is believed that abdominal pregnancy is more common in developing countries probably because of the high frequency of pelvic inflammatory disease in these areas.[2] Heterotopic pregnancy is the coexistence of intrauterine and extra-uterine pregnancies. Abdominal pregnancies make up a small percentage of ectopic pregnancies which are a common occurrence.[3] Moreover, 98% of all extra-uterine pregnancies are intra-tubal, one percent is ovarian and the rest are primary or secondary peritoneal implantations. Atrash and colleagues estimated the incidence of abdominal pregnancy at 10.9 per 100,000 live births and 9.2 per 1,000 ectopic pregnancies in the United States.[1]

Abdominal pregnancies can be classified as ‘primary’ when fertilization takes place outside the uterine adnexae, or as ‘secondary’ (thought to be more common) believed to result from undetected rupture of a tubal pregnancy. Implantation can occur anywhere in the abdomen including ligaments, liver, and spleen. Recent estimated rate of occurrence of heterotopic pregnancy is 1 in 15,000 live births and the ectopic component is commonly tubal. Its incidence is increased in women undergoing assisted conception with superovulation, IVF-ET and GIFT.[4]

## CASE REPORT

A 26-year-old primigravida presented to our emergency surgical department as a case of pain in right lower abdomen of two days duration with history of three months amenorrhea. The patient had 2-3 episodes of vomiting and was febrile from last one day. She gave a history of giddiness on standing. On examination, she was conscious and oriented with pulse of 98 beats per minute and BP of 90/65 mmHg. Her abdomen was mildly distended and tender in the right iliac fossa (RIF). A tender lump about 10 × 12 cms was found in her RIF. Based on the history and clinical examination, the patient was clinically diagnosed as a case of ruptured ectopic pregnancy.

**Figure 1:**
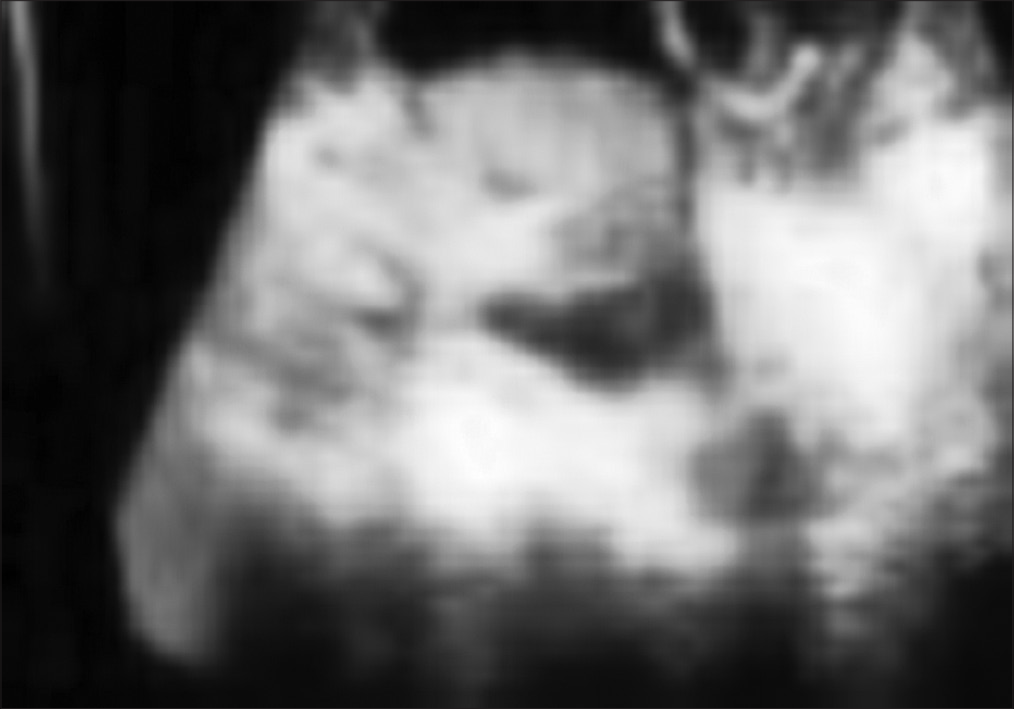
Ultrasonography showing intra-uterine pregnancy

**Figure 2:**
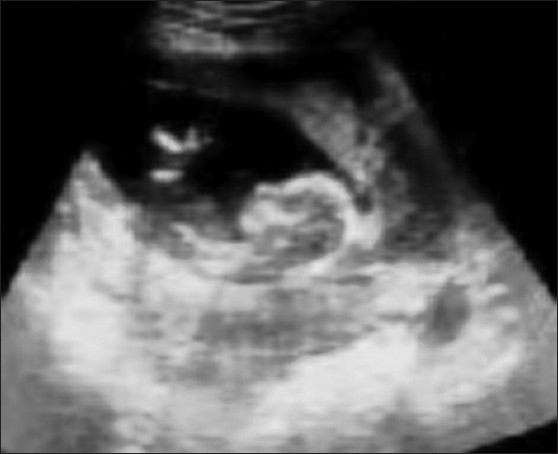
Ultrasonography showing intra-abdominal pregnancy

**Figure 3:**
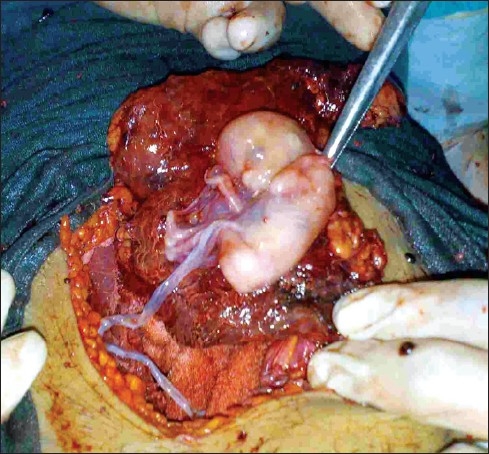
Intra-abdominal fetus being delivered

The patient was immediately resuscitated with I.V. fluids and a urinary catheter was put in to monitor the output. Subsequently, an ultrasonographic (USG) examination was conducted which diagnosed her as a case of heterotopic pregnancy; with intrauterine pregnancy [Figure 1] and the other one in the abdomen, implanted on the iliocolic region [Figure 2] of 13 weeks duration. USG also showed the presence of significant free fluid in the peritoneal cavity (hemoperitoneum). She was immediately prepared for emergency laparotomy under general anesthesia. Abdomen was opened by lower midline incision. On opening the abdomen, about 1.5 liters of free blood was found in the peritoneal cavity. The intra abdominal pregnancy was confirmed; and the partially detached placenta was seen implanted over the illiocolic region, leading to the hemoperitoneum. The fetus was delivered and the placenta was meticulously separated [Figure 3]. Intrauterine pregnancy was not disturbed. Complete hemostasis was achieved and abdomen was closed. Her postoperative course was unremarkable. She was discharged on 5^th^ postoperative day with no surgical or any other postoperative complication.

The patient was followed till term and delivered a normal female baby by caesarean section due to the persistent oblique position of the fetus confirmed by USG examination. There were no postoperative complications and the patient was discharged without any untoward event.

## DISCUSSION

Heterotopic pregnancy is a potentially fatal disease. Incidence of heterotopic pregnancy has been reported as 1/8000-1/30,000 in natural conception.[5] It may increase as high as 1% with assisted reproductive techniques. Diagnosis of heterotopic pregnancy is a challenge not only for the obstetricians but also for other physicians who are following or treating the patient.

Spontaneous progression of undetected intrauterine pregnancy from the time of surgical management of acute or subacute ruptured ectopic pregnancy on postoperative follow up is rare. On the contrary, spontaneous abortion of an intrauterine pregnancy has followed ectopic rupture.[6]

Risk factors include a history of tubal pregnancies, pelvic inflammatory disease, tubal sterilization, and tubal infertility or tubal reconstructive surgery. Other women at risk include those who conceive despite the use of an intra- uterine contraceptive device (IUCD) or progestogen only contraceptive pills.[7] Since none of the above risk factors were present, the undetected rupture of a tubal pregnancy was considered as case of heterotopic pregnancy in our case.

Early diagnosis depends on the clinician having a high index of suspicion. Reece *et al.*[8] defined four common symptoms and findings. These are; abdominal pain, adnexal mass, peritoneal irritation, and increase in the size of the uterus. While Tal *et al.*, reported abdominal pain in 83% and abdominal tenderness with hypovolemic shock in 13% of the heterotopic pregnancy cases and vaginal bleeding in half of the patients. Finding of vaginal bleeding that can be concurrent in ectopic pregnancies is rarely seen in heterotopic pregnancies on account of intact endometrium of intrauterine pregnancy.[9] Quantitative measurements of serum beta human chorionic gonadotropin (β-HCG) levels are of no use, because the intra-uterine pregnancy will be producing normal and increasing levels of serum β-HCG.[10]

Abdominal pregnancy poses a serious threat to the survival of equally the mother and the fetus. Hence it is vital that the diagnosis is made early in the pregnancy. Maternal mortality ranges between 0 and 30 percent.[11] This is principally because of the risk of massive hemorrhage from incomplete or entire placental separation. The placenta can be attached to the uterine wall, bowel, mesentery, liver, spleen, bladder, and ligaments, which can separate at anytime during pregnancy leading to heavy blood loss. The fetal outcome tends to be poorer than the mother's with perinatal mortality ranging between 40 and 95 percent.[12] Fetal abnormalities are also high with a number of congenital malformations being common. However, with advanced pregnancy and if the fetus is surrounded by a normal volume of amniotic fluid, fetal outcome tends to be better.[11]

The diagnosis of abdominal pregnancy is complex. Ultrasound, when coupled with clinical evaluation, has approximately a 50 percent success rate in the diagnosis.[3] Allibone *et al.*[13] have provided guidelines for the use of USG to diagnose abdominal pregnancy; the reported diagnostic errors in different series have ranged from 50 to 90%. In our case, USG was able to accurately diagnose this condition. An MRI scan can also be used to confirm the diagnosis of abdominal pregnancy. Laboratory tests such as abnormally increasing human chorionic gonadotrophin, are not sufficiently reliable on their own to make a diagnosis, as are signs and symptoms such as the abdominal pain and tenderness, persistent transverse or oblique lie and palpable fetal parts.[3]

For the management of abdominal pregnancy, factors such as maternal hemodynamic status, fetal congenital abnormality, fetal viability, gestational age at presentation, and the availability of neonatal facilities should be considered. If the fetus is dead, surgical intervention is generally indicated owing to the risk of infection and disseminated intravascular coagulation. Various clinicians, however, recommend a period of observation of 3 to 8 weeks to allow atrophy of placental vessels to occur.[14] If the fetus is alive, laparotomy should be performed, regardless of gestational age or foetal condition.[2] The reason is mainly based on the unpredictability of placental separation and consequential massive haemorrhage.

## CONCLUSION

To conclude, our case exemplifies the inclusion of heterotopic pregnancy in the causes of hemoperitoneum in young females since this condition is often forgotten due to its rarity and atypical presentations. As such high index of suspicion is required for the salvage of the intrauterine pregnancy as well as for the accurate diagnosis at an early stage before fatal complications occur.
